# YWHA (14-3-3) protein isoforms and their interactions with CDC25B phosphatase in mouse oogenesis and oocyte maturation

**DOI:** 10.1186/s12861-019-0200-1

**Published:** 2019-10-22

**Authors:** Alaa A. Eisa, Santanu De, Ariana Detwiler, Eva Gilker, Alexander C. Ignatious, Srinivasan Vijayaraghavan, Douglas Kline

**Affiliations:** 1 School of Biomedical Sciences, Kent State University, Kent, OH 22422 USA; 2 Department of Biological Sciences, Nova Southeastern University, Fort Lauderdale, FL 33314 USA; 3 Department of Environmental and Occupational Health, University of Pittsburgh Graduate School of Public Health, UPMC Hillman Cancer Center, Pittsburgh, PA 15213 USA; 4 Department of Physiology and Biophysics, Case Western Reserve University School of Medicine, Cleveland, OH 44106 USA; 5 Department of Biological Sciences, Kent State University, Kent, OH 44242 USA

**Keywords:** Meiosis, Oogenesis, Oocyte maturation, YWHA, 14-3-3, YWHAH, YWHAE, CDC25B, Mouse

## Abstract

**Background:**

Immature mammalian oocytes are held arrested at prophase I of meiosis by an inhibitory phosphorylation of cyclin-dependent kinase 1 (CDK1). Release from this meiotic arrest and germinal vesicle breakdown is dependent on dephosphorylation of CDK1 by the protein, cell cycle division 25B (CDC25B). Evidence suggests that phosphorylated CDC25B is bound to YWHA (14-3-3) proteins in the cytoplasm of immature oocytes and is thus maintained in an inactive form. The importance of YWHA in meiosis demands additional studies.

**Results:**

Messenger RNA for multiple isoforms of the YWHA protein family was detected in mouse oocytes and eggs. All seven mammalian YWHA isoforms previously reported to be expressed in mouse oocytes, were found to interact with CDC25B as evidenced by in situ proximity ligation assays. Interaction of YWHAH with CDC25B was indicated by Förster Resonance Energy Transfer (FRET) microscopy. Intracytoplasmic microinjection of oocytes with R18, a known, synthetic, non-isoform-specific, YWHA-blocking peptide promoted germinal vesicle breakdown. This suggests that inhibiting the interactions between YWHA proteins and their binding partners releases the oocyte from meiotic arrest. Microinjection of isoform-specific, translation-blocking morpholino oligonucleotides to knockdown or downregulate YWHA protein synthesis in oocytes suggested a role for a specific YWHA isoform in maintaining the meiotic arrest. More definitively however, and in contrast to the knockdown experiments, oocyte-specific and global deletion of two isoforms of YWHA, YWHAH (14-3-3 eta) or YWHAE (14-3-3 epsilon) indicated that the complete absence of either or both isoforms does not alter oocyte development and release from the meiotic prophase I arrest.

**Conclusions:**

Multiple isoforms of the YWHA protein are expressed in mouse oocytes and eggs and interact with the cell cycle protein CDC25B, but YWHAH and YWHAE isoforms are not essential for normal mouse oocyte maturation, fertilization and early embryonic development.

## Introduction

Oocytes are held in the first meiotic prophase within the mammalian ovary. Prophase arrest is dependent on the production of cAMP within the oocyte. Constitutively active heterotrimeric G protein receptors linked to stimulatory G proteins activate adenylyl cyclase to maintain a high concentration of cAMP within the oocyte [1–3] reviewed by Jaffe and Egbert [4]. Meiotic arrest is maintained by cAMP-dependent activation of protein kinase A (PKA), in part, by phosphorylating and activating the oocyte-specific kinase WEE2 (also known as WEE1B) [5–8]. WEE2, in turn, is thought to phosphorylate and inactivate cyclin-dependent kinase 1 (CDK1), which together with regulatory cyclin B1 (CCNB1), forms maturation promoting factor (MPF) reviewed in [9, 10]. MPF is well characterized, based on studies of oocytes of many species as well as somatic cells, since it is the cell cycle regulatory factor for both meiotic and mitotic cells reviewed in [11–13]. Inhibitory phosphorylation of CDK1 maintains MPF in an inactive state within the oocyte. PKA is also thought to phosphorylate cell division cycle 25B (CDC25B) phosphatases [14–16], keeping CDC25B proteins in an inactive state that preserves the phosphorylated and inactive status of CDK1 a target of CDC25B. In the arrested mouse oocyte, genetic studies have shown that CDC25B, and not CDC25C, is the primary phosphatase regulating the dephosphorylation of CDK1, [17, 18] although CDC25A also appears to play a role in oocyte maturation [19].

The high level of cAMP in the follicle-enclosed oocyte, generated by the oocyte itself, is sustained by cGMP produced in granulosa cells, which passes through gap junctions and inhibits the hydrolysis of cAMP by the cAMP phosphodiesterase, PDE3A [20]. Oocytes in pre-ovulatory follicles resume meiosis in response to luteinizing hormone (LH) acting on granulosa cells. LH initiates multiple signaling pathways including the interruption of the flow of inhibitory cGMP by inactivation of the NPR2 guanylyl cyclase and stimulation of cGMP hydrolysis by activation of a phosphodiesterase [4]. Activation of the cAMP phosphodiesterase within the oocyte reduces cAMP level and PKA activity. MPF is activated following reduced phosphorylation by WEE2 and the dephosphorylation of CDK1 by the protein phosphatase, CDC25B, reviewed in [10]. Activation of MPF initiates the resumption of meiosis and the process of oocyte maturation beginning with nuclear envelope breakdown (germinal vesicle breakdown or GVBD) followed by formation of the first polar body and, in most mammals, arrest at metaphase of the second meiotic division, reviewed in [21, 22]. Oocyte maturation forms the mature egg and fertilization releases the egg from metaphase II arrest, allowing formation and development of the zygote.

The phosphorylation and activity of CDC25 proteins appears to be preserved, in part, by binding to YWHA regulatory proteins (Tyrosine 3-Monooxygenase/Tryptophan 5-Monooxygenase Activation protein or 14-3-3). YWHA proteins are thought to both maintain the phosphorylated status of CDC25 and sequester CDC25 in the oocyte cytoplasm. Early studies demonstrated that, in *Xenopus* oocytes, CDC25 phosphatase is phosphorylated by PKA, and is bound to and sequestered by YWHA in the cytoplasm [23], thus maintaining the cell cycle arrest. Numerous studies implicate YWHA as a critical regulator of the cell cycle in meiotic and mitotic cells as well as other cellular processes [24–35]. The YWHA proteins also have multiple binding partners in mammalian testes and sperm [36, 37]. A YWHA protein also appears to bind to and likely regulate peptidyl arginine deiminase type VI (PADI6) in mice and humans [38, 39].

The YWHA proteins are a highly conserved, homologous family of proteins shown to bind to various cellular proteins and complement or supplement intracellular events involving phosphorylation-dependent switching or protein-protein interaction [33, 40]. Most of the binding partners of YWHA are phosphorylated; however, some interactions of YWHA do exist independent of phosphorylation [41]. The YWHA proteins exist mainly as homo- or hetero-dimers with a monomeric molecular mass of approximately 30 kDa [33]. There are seven mammalian isoforms of YWHA encoded by separate genes: *Ywhab* (14-3-3β), *Ywhae* (14-3-3ε), *Ywhah* (14-3-3η), *Ywhag* (14-3-3γ), *Ywhaz* (14-3-3ζ), *Ywhaq* (14-3-3τ/θ) and *Sfn* (14-3-3σ).

Using isoform-specific antibodies, we found that all seven mammalian isoforms of YWHA are expressed in mouse ovaries, immature oocytes and mature eggs [42]. In contrast, one report indicated that only YWHAB and YWHAE are present in mouse oocytes [43]. This was surprising since our panel of antibodies had identified more isoforms and, for example, transcripts of at least six isoforms of *Ywha* are present in mouse eggs [44] and all seven *Ywha* isoform messages are found in human eggs [45, 46]. In this report, we include additional evidence for the presence of *Ywha* mRNA for seven isoforms of YWHA in two different mouse strains.

It is known that different isoforms of YWHA can interact with the same ligand and so are somewhat interchangeable; however, although isoforms of YWHA often bind the same protein, there are some indications that homodimers of different types or even heterodimers of YWHA may have different roles in the regulation or sequestering of proteins [41]. Therefore, it is important to determine which YWHA isoform(s) interact(s) with CDC25B in the oocyte to maintain the meiosis I arrest. We examined YWHA-CDC25B interactions using in situ Proximity Ligation Assay (PLA) and Förster Resonance Energy Transfer (FRET) approaches.

We performed experiments to inhibit YWHA interactions with target proteins by injection of the YWHA-inhibitory peptide, R18. In exploratory work shown here, we aimed to reduce the expression of specific YWHA proteins by intracellular microinjection of a translation-blocking morpholino oligonucleotide against each of the YWHA isoform mRNAs. To definitively clarify a role of YWHAH and YWHAE, we generated oocyte-specific and global knockout mice in which genes for *Ywhah*, *Ywhae*, or both, were disrupted. Other previous reports of global *Ywha* knockouts of different isoforms have given limited information about oogenesis. The *Ywhag* knockout shows no abnormal phenotype, although it is not clear that reproductive potential was examined in detail [47]. With complete germline deletion of *Ywhae* in a global knockout, mice die at birth, due to cardiac malformations or other causes [48, 49] with prenatal embryos showing hippocampal and cortical defects in the brain [49]. *Ywhaz* knockout mice also show neurological defects [50, 51].

The experiments outlined in this work provide evidence that multiple YWHA isoforms interact with CDC25B in mouse oocytes and mature eggs and report for the first time on mice with two of the *Ywha* genes inactivated. The results indicate that oocyte-specific or global elimination of YWHAH protein does not result in abnormal fertility, oocyte maturation or development. Oocyte-specific or global elimination of YWHAE does not alter oocyte maturation*,* in vitro fertilization and early development, though global inactivation of *Ywhae* does appear to impair in vivo fertility.

## Results

### Detection of mRNAs for *Ywha* isoforms in mouse oocytes and eggs

**Fig. 1:**
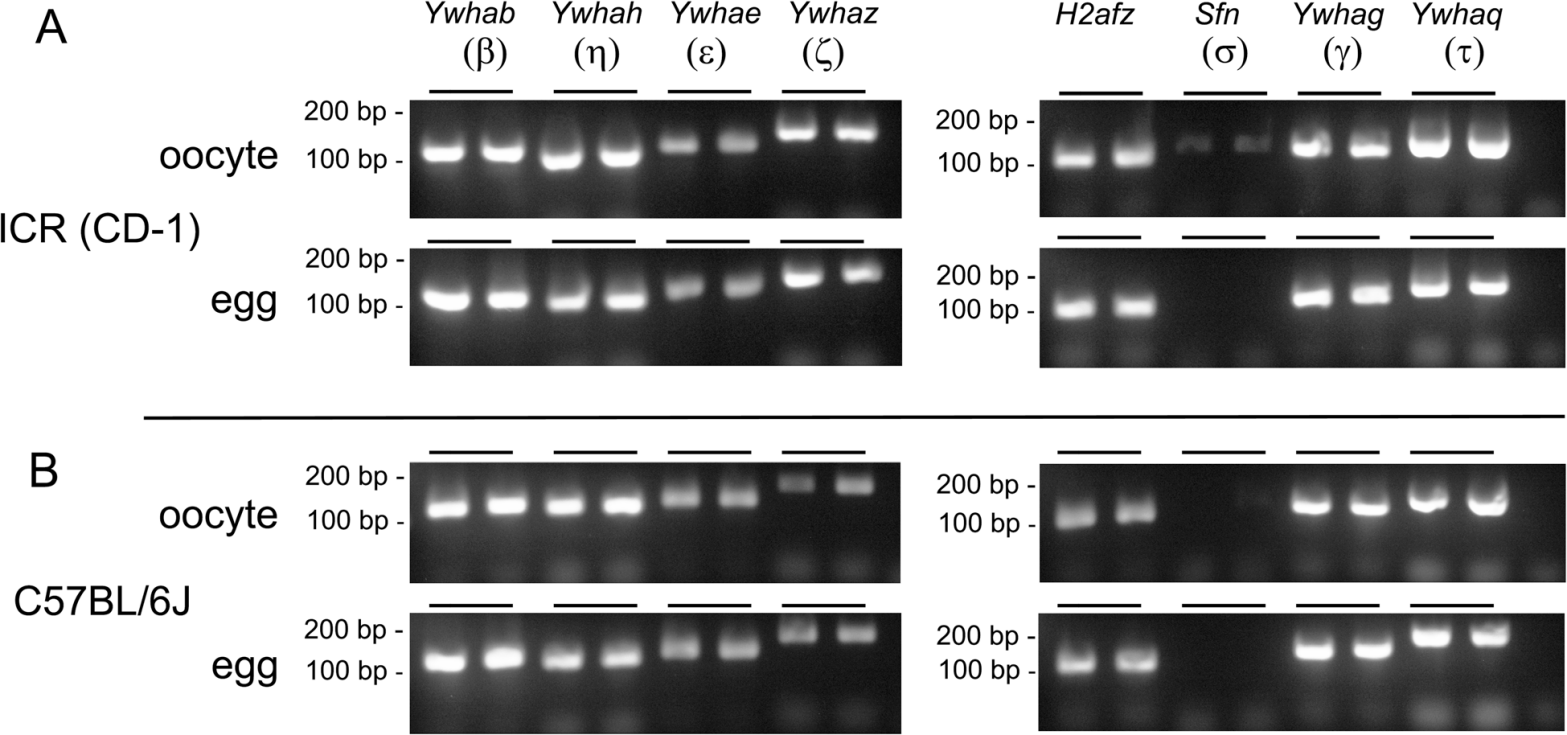
Detection of mRNAs for *Ywha* isoforms in oocytes and eggs of two mouse strains. Messenger RNA was isolated from oocytes and eggs, reverse transcribed and prepared for PCR as indicated in Methods. The PCR reactions using isoform-specific *Ywha* primers were repeated twice for each isoform and the products were loaded in adjacent lanes. The upper panel (**a**) displays PCR products of extracts of oocytes and eggs from outbred ICR (CD-1) mice. The lower panel (**b**) displays PCR products of extracts of oocytes and eggs from inbred C57BL/6 J mice. *H2afz* served as a positive PCR control. In all cases, no bands were seen in the negative controls consisting of all the PCR reaction mixture components excluding the template. Message corresponding to six *Ywha* isoforms was detected in both strains. The signal for *Sfn* message was barely detectable in oocytes of ICR(CD-1) oocytes

It has been reported earlier that all seven YWHA proteins are expressed in mouse oocytes and eggs, based on the use of isoform-specific antibodies [42]. To confirm this observation, the presence of mRNA for all the isoforms was examined in both oocytes and eggs. Isoform-specific primers were used to amplify the messages for the seven isoforms by RT-PCR (Fig. 1). Messages for six of the seven mammalian isoforms were found in oocytes of both outbred ICR(CD-1) and inbred C57BL/6 J strains. Message for the seventh isoform, *Sfn* (14-3-3σ) was just detected in oocytes of ICR(CD-1) mice, but it was not detected in the C57BL/6 J strain. In a parallel experiment, we sequenced the RT-PCR products using specific forward and reverse primers. We obtained sequence data for *H2afz* mRNA and all isoforms of *Ywha* except *Sfn*, as the product yield for *Sfn* cDNA was too low for accurate sequencing. As expected, 50–60% of the expected short PCR product was reliably sequenced and for each sequence the valid base calls exactly matched the NCBI mouse mRNA RefSeq sequences.

We also examined existence of the *Ywha* transcripts in mouse oocytes of ICR(CD-1) wild-type mice and several knockout models by mRNA sequencing of single oocytes (Table 1). Reads from RNA-seq were annotated to Ensembl. FPKM (fragments per kilobase of exon model per million reads mapped), the normalized estimation of gene expression based on the RNA-seq data, was estimated using Cufflinks. Messages for all seven isoforms of *Ywha* were detected (though *Sfn* is not as abundant). While the data is based only on one cell of each type, the FPKM values are similar among five cells examined, expect for those cells in which *Ywhah* and/or *Ywhae* genes were inactivated.

**Table 1:** Detection of *Ywha* isoform mRNAs in single oocytes

	YWHAH	YWHAE	YWHAQ	YWHAZ	YWHAB	YWHAG	SFN
Wild type	63.7	365.1	75.1	132.5	58.9	23.4	2.7
*Ywhae* CKO	61.7	(13.1)	52.4	96.8	44.5	23.4	4.3
*Ywhah* CKO	(0.6)	323.7	87.3	126.3	47.8	19.5	7.6
*Ywhae* and *Ywhah* CKO	(0.5)	(18.3)	119.0	97.5	48.1	13.3	7.6
*Ppp1cc* KO	44.6	309.2	47.8	89.8	35.5	5.6	1.8
Mean	56.7 ± 10.5	332.7 ± 29.0	76.4 ± 28.8	108.6 ± 19.4	47.0 ± 8.4	14.6 ± 7	4.8 ± 2.7
	*n* = 3	*n* = 3	*n* = 5	*n* = 5	*n* = 5	*n* = 5	*n* = 5

FPKM values for *Ywha* isoform transcripts in single oocytes were obtained from an ICR(CD-1) wild-type female, and from females with the *Ywhae* oocyte-specific knockout (CKO), *Ywhah* CKO, the oocyte-specific double CKO for *Ywhae* and *Ywhah*, and an oocyte from a female in which the *Ppp1CC* gene is globally inactivated (see Methods for details on the generation of these animals). The mean FPKM (with standard deviation) for each gene product is indicated utilizing all values except those in parentheses (CKO oocytes). Ensembl transcript IDs (mouse version GRCM38.p6): Ywhah, ENSMUST00000019109; Ywhae, ENSMUST00000067664; Ywhaq, ENSMUST00000135088; Ywhaz, ENSMUST00000022894; Ywhab, ENSMUST00000018470; Ywhag, ENSMUST00000055808; Sfn, ENSMUSG00000047281.

### YWHAH and YWHAE protein distribution in the mouse oocyte

**Fig. 2:**
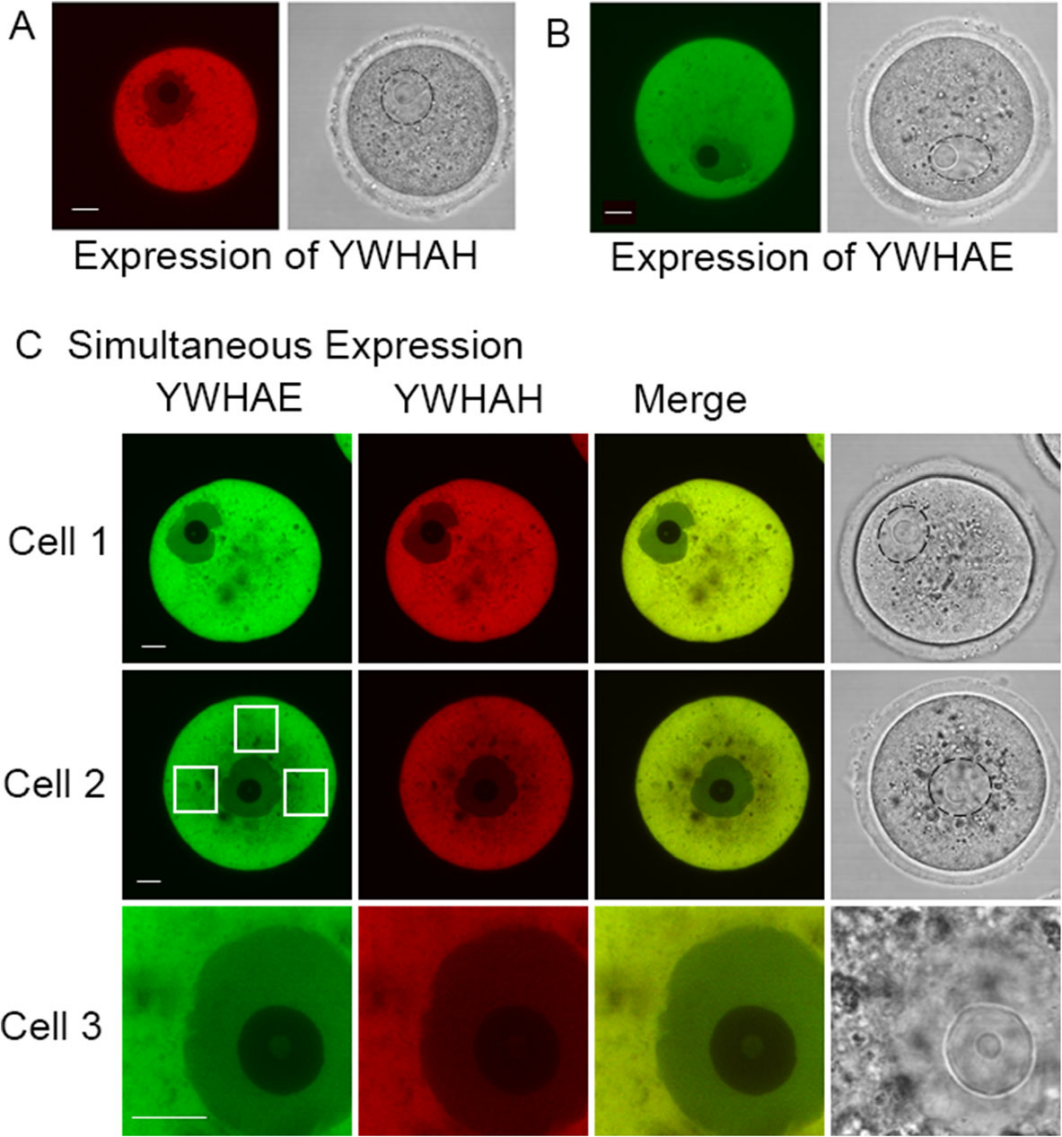
Expression of fluorescently-tagged YWHAH and YWHAE in mouse oocytes. Immature oocytes from wild type ICR(CD-1) mice were microinjected with mCherry-YWHAH (**a**) or EGFP-YWHAE mRNA (**b**). The corresponding brightfield images are shown at the right and the oocyte nucleus is outlined with a dashed black line. The images are representative of results from 16 different oocytes expressing mCherry-YWHAH from five different experiments and 12 oocytes expressing EGFP-YWHAE from three different experiments. **c** Representative images of three oocytes simultaneously injected with mCherry-YWHAH and EGFP-YWHAE mRNA. Both isoforms are expressed in the same cell and the merged emission channels suggests a very similar distribution for both isoforms. The white squares shown in Cell 2 are the regions of interest used to determine the average Pearson’s correlation coefficient for this cell (see Results). The nucleus was imaged at higher magnification in Cell 3. There is a clear distinction between the cytoplasm and the nucleus, with the nucleus displaying less fluorescence than the cytoplasm and the nucleolus displaying little fluorescence. YWHAH and YWHAE are primarily cytoplasmic in an immature oocyte. All of seven other injected cells were similar. The scale bars represent 10 μm

A previous study determined that the distribution of YWHA isoforms in the cytoplasm and in the nucleus varied, depending on the isoform [42]. Because YWHA proteins are thought to sequester CDC25B in the cytoplasm of immature, prophase I-arrested oocytes, we investigated the distribution of two isoforms of interest, YWHAH and YWHAE by an alternative approach. We designed mRNA constructs that, after mRNA injection and expression in mouse oocytes, produced fluorescently labeled proteins, mCherry-YWHAH and EGFP-YWHAE. Oocytes expressed both fluorescent proteins very efficiently which, for each isoform, was primarily in the cytoplasm and much less so in the nucleus (Fig. 2a, b).

Expression of mCherry-YWHAH and EGFP-YWHAE following co-injection of both mRNAs indicates that these two proteins co-localize to some extent as there was spatial overlap of signal from the two separate emission wavelengths. The emission spectra of these two fluorescent probes display a clear separation of the peak excitation and emission wavelengths, thus there is very low crosstalk between the two excitation/emission channels. In addition, we use sequential confocal scanning to prevent overlap of excitation and emission wavelengths. Digitally combining the two images from sequential scans recording emissions from mCherry-YWHAH and EGFP-YWHAE reveals a similar distribution of the two fluorescence signals throughout the oocyte (Fig. 2c). Further analysis, in the form of a scatter plot and analysis of pixel values along with determination of Pearson’s correlation coefficient revealed some colocalization. In Pearson’s correlation, the average pixel intensity values are subtracted from the original intensity values, so the value of this coefficient ranges from − 1 to 1, with a value of − 1 representing a total lack of overlap between pixels from the images, and a value of 1 indicating perfect image correspondence. Analysis of the optical image of Cell 2 in Fig. 2c, for example, revealed an average of Pearson’s correlation coefficient, r, over three regions of interest was 0.31 indicating a considerable degree of colocalization.

### CDC25B expression in the oocyte

**Fig. 3:**
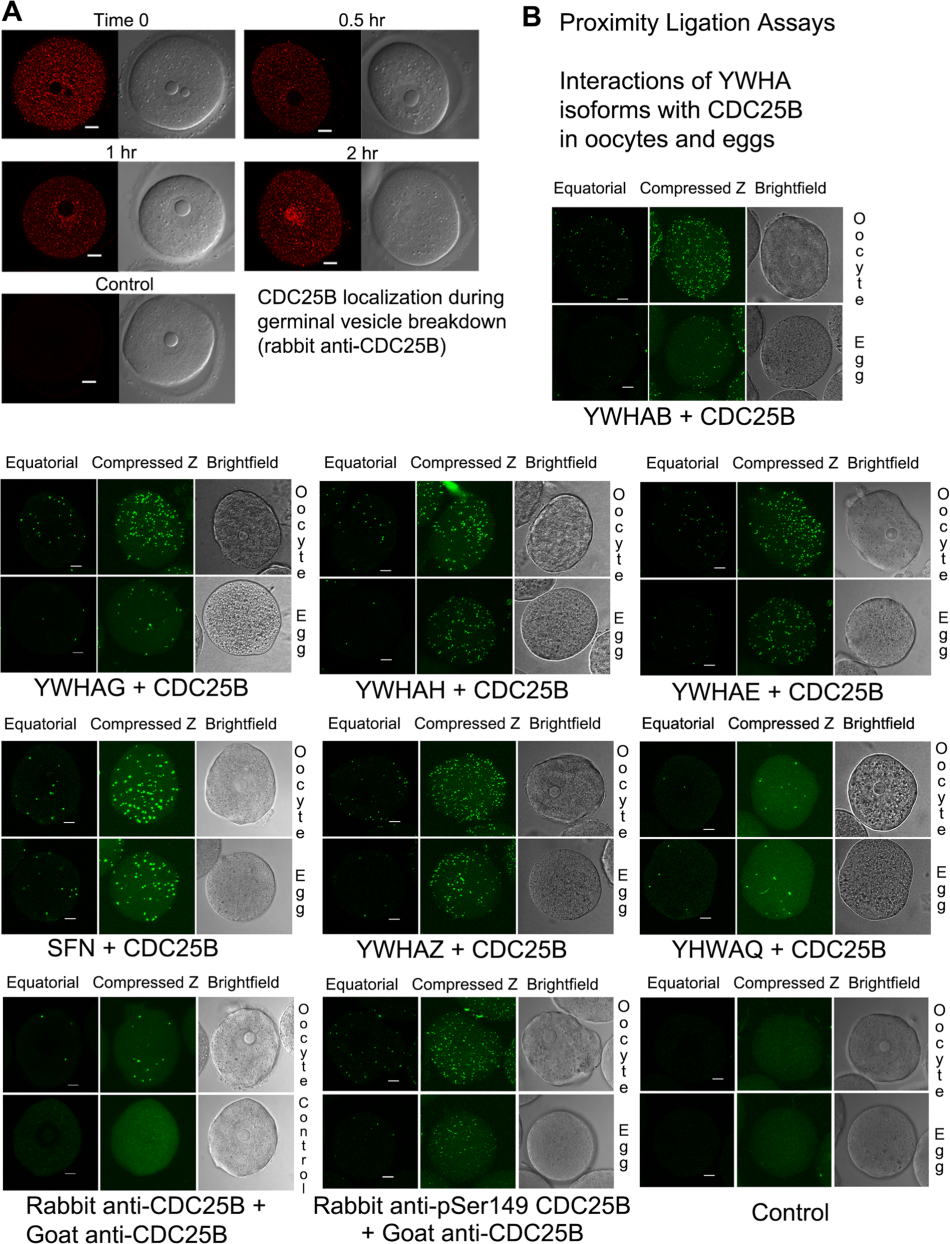
CDC25B distribution during oocyte maturation and interactions with YWHA proteins demonstrated by proximity ligation assays in oocytes and eggs. **a** Paired confocal images of equatorial sections through oocytes (left: immunofluorescence; right: brightfield) with rabbit anti-CDC25B. CDC25B is present thorught the oocyte and accumulates in the region of the nucleus across a two-hour time course during in vitro maturation of mouse oocytes. Control oocytes processed simultaneously in absence of the primary antibody showed minimal background fluorescence. These images are representative of two experiments that examined 6–12 cells at each time point. **b** Representative images of oocytes and eggs showing proximity ligation reaction spots that indicate interaction of YWHA protein isoforms with CDC25B at the molecular level. Each reaction spot represents a protein-protien interacton between a CDC25B and a YWHA protein (see Methods for details). Except for the lower left images, the upper panel is an oocyte and the lower panel is an egg, each imaged with confocal fluorescence microscopy. The equatorial scan is a single scan and the compressed Z is obtained from combining consecutive cross-sectional images throughout each cell taken at 3 μm intervals. The corresponding brightfield image is included. The lower two left panels are representative of the experiment using PLA with different primary antibodies against CDC25B directed at different sites on the protien. Binding to the same protein is indicated by PLA spots (rabbit anti-CDC25B and goat anti-CDC25B; rabbit anti-pSer149 CDC25B and goat anti-CDC25B). No reaction spots are observed in the control (oocytes and eggs processed in absence of the primary antibodies). The images shown are representative of two experiments (oocytes and eggs from four animals) with similar results for a total of 7–10 oocytes and a total of 6–11 eggs for each condition and controls. Scale bars for all images represent 10 μm

Immunofluorescence confocal imaging reveals that CDC25B is distributed throughout the prophase I-arrested oocyte cytoplasm and some CDC25B is also present within the nucleus (Fig. 3a). During the initial stage of oocyte maturation, CDC25B accumulates in the nucleus and by 2 h after release from meiotic arrest, the concentration of CDC25B is much greater in the region where the nucleus had undergone GVBD. In all oocytes studied at each time point, the identical distribution of CDC25B was noted. Minimal background staining was observed in control oocytes processed simultaneously and imaged at the same confocal settings, but without the primary antibody (Fig. 3a).

### In situ proximity ligation assays (PLA) reveal interaction of CDC25B with all isoforms of YWHA in oocytes and eggs

To determine the possible interactions between each of the YWHA isoforms and CDC25B, we conducted a series of in situ proximity ligation assays (PLA) that identify interactions of two proteins at the molecular level by fluorescent detection probes. The PLA method has been used successfully to detect protein-protein interactions at the single molecule level directly in cells, and to visualize the actual intracellular sites of the interactions in different types of cells and tissues [52–55]. The PLA assay revealed interactions of all seven YWHA protein isoforms with CDC25B within mouse oocytes and eggs using primary antibodies directed at each of the YWHA isoforms and CDC25B. Representative equatorial as well as compressed Z-stack images of oocytes and eggs from PLA assays are provided in Fig. 3b. Interactions are visible as fluorescent spots in a single equatorial scan in most cases. Each cell was scanned at 3 μm intervals throughout the cell. The abundance of interaction sites is visualized when these scans are compressed into one image (compressed Z in Fig. 3b). It should be noted that the PLA method does not show the complete distribution of all CDC25B and YWHA proteins as would be shown in conventional immunofluorescence, but does show specific protein-protein interactions when a number of criteria have been met that include binding of the two primary antibodies to the target proteins, binding of secondary probes, ligation, amplification and hybridization of fluorescent detection probes. The PLA technique is sensitive, specific, and provides a high signal to noise ratio because the signal is amplified, and close proximity of the target proteins is required (< 40 nm critical distance). Each of these steps is concentration- and time-dependent. The number of fluorescent spots represents only a portion of the protein-protein interactions. However, this method clearly provides more information about direct protein-protein interactions than simple colocalization studies with immunofluorescence microscopy. It is not possible to compare the results (the number of interaction sites, for example) among the different YWHA isoforms because of differences in primary antibody binding affinities.

No control oocytes or eggs, treated simultaneously and under identical conditions, incubated either with no primary antibodies (Fig. 3b, control), or with goat anti-CDC25B primary antibodies alone, or with rabbit anti-YWHA primary antibodies alone, displayed any PLA reaction spot. Background fluorescence from unbound fluorescent detection probes was minimal. To confirm the effectiveness of the PLA method, we processed oocytes and eggs for PLA using the goat anti-CDC25B with two different antibodies to CDC25B generated against different isotopes on the same protein (a general rabbit antibody against CDC25B and a rabbit antibody against p-Ser 149 CDC25B). Detection of the PLA reaction spots with antibodies bound to the same protein provides evidence for the effectiveness of the PLA method as the primary anitbodies should be well within 40 nm of each other to enable secondary probe DNA ligation and amplification. With these two antibodies, in situ PLA reaction spots were detected in all oocytes examined (a representative exmple is shown in Fig. 3b).

### FRET analysis indicates close interaction of YWHAH with CDC25B

We utilized fluorescently tagged YWHAH and CDC25B to further examine possible interaction between these two proteins. Messenger RNA for mCherry-YWHAH and EGFP-CDC25B was introduced by microinjection into oocytes and, following overnight incubation, expression of the proteins was analyzed by confocal microscopy and Förster Resonance Energy Transfer (FRET). Cells microinjected with only donor (EGFP-CDC25B) mRNA or only acceptor (mCherry-YWHAH) mRNA were imaged, along with the FRET samples co-injected with messages for both proteins. Under normal conditions, EGFP is excited by blue light (488 nm) and emits green fluorescence (507 nm). However, during FRET by sensitized emission, the green emission would transfer some energy to excite the mCherry fluorophores if they are within the Förster distance (5.1 nm) and red fluorescence (610 nm) will be emitted.

**Fig. 4:**
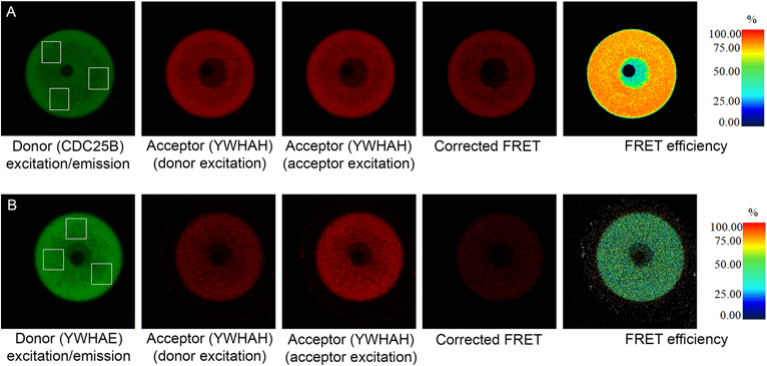
FRET analysis of interaction of YWHAH with CDC25B and YWHAH with YWHAE. **a** ICR(CD1) oocytes were co-injected with mCherry-YWHAH and EGFP-CDC25mRNA. Expression of EGFP-CDC25B protein provides the donor fluorescence (green) in the FRET analyses. The mCherry-YWHAH serves as the acceptor fluorescent probe (red). Images show the donor emission with donor excitation (488 nm), the acceptor emission with donor excitation, and the acceptor emission with acceptor excitation (543 nm), and the corrected FRET image. Data from three regions of interest (shown by white boxes) were used to calculate the FRET efficiency, which, in this cell averaged 72%, and is visually represented in the heat map at the right. The figure is representative of 12 oocytes collected from three different females in three experiments. **b** ICR(CD1) oocytes were co-injected with mCherry-YWHAH and EGFP-YWHAE. EGFP-YWHAE protein provides the donor fluorescence (green) in the FRET analyses. The mCherry-YWHAH serves as the acceptor fluorescent probe (red). Images show the donor emission with donor excitation (488 nm), the acceptor emission with donor excitation, and the acceptor emission with acceptor excitation (543 nm), and the corrected FRET image. Data from three regions of interest (show by white boxes) was used to calculate the FRET efficiency, which in this cell averaged 27%, and is visually represented in the heat map at the right. The figure is representative of five oocytes examined in two experiments

FRET efficiency was measured in three different regions of interest in the cytoplasm for each oocyte and average of these measurements was determined (Fig. 4). The range of FRET efficiency ranges from 64 to 82% (*n* = 12), averaging 73% for all cells. This strong FRET efficiency suggests a close interaction between YWHAH and CDC25B protein, which is consistent with the Duolink analyses. We have not examined other isoforms by FRET interactions but might expect a similar result. In comparison, we also examined the interaction of mCherry-YWHAH and EGFP-YWHAE proteins (Fig. 4). The range of FRET efficiency ranges from 25 to 27% (*n* = 5), averaging 22.5% for all cells. This is a fairly strong interaction and might be suggestive of heterodimer formation between YWHAE and YWHAH, which would need to be examined further.

### Microinjection of oocytes with the YWHA-inhibitory peptide R18 promotes oocyte maturation

As detailed in the Introduction, it is thought that CDC25B is phosphorylated and that CDC25B is held in an inactive state by being bound to YWHA proteins. Therefore, to demonstrate directly in mouse oocytes that YWHA/CDC25B interaction is required for meiotic arrest, we performed a series of experiments to disrupt this interaction, with the hypothesis that preventing the interaction of CDC25B with YWHA proteins would lead to oocyte maturation by rendering the CDC25B free to dephosphorylate CDK1 and activate MPF.

**Fig. 5:**
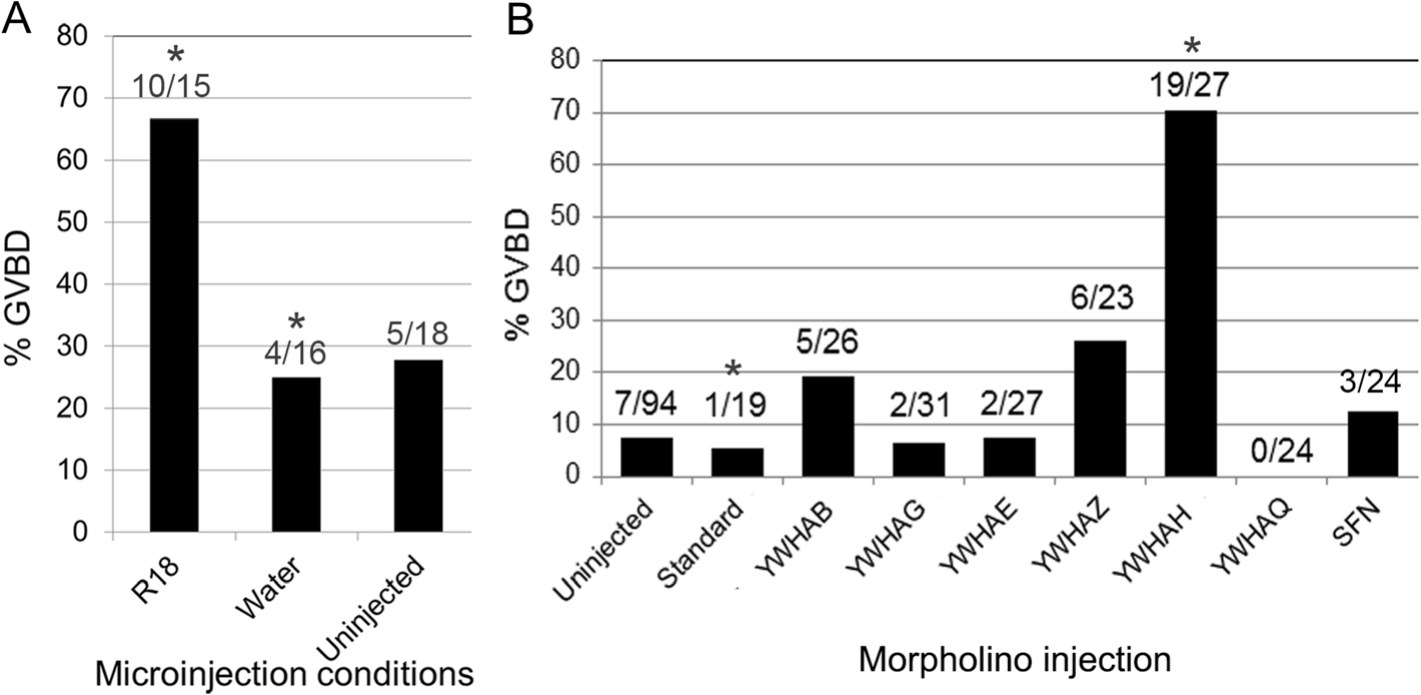
Injection of the YWHA-inhibitory peptide and *Ywha* mRNA antisense morpholinos against mRNA for each *Ywha* isoform. For each injection condition and the uninjected controls in this series of experiments, the number of cells with GVBD is represented as a percent of the total number of cells studied (indicated at the top the corresponding bar). See the Methods section for experimental methods. **a** Percent germinal vesicle breakdown (GVBD) in mouse oocytes injected with the YWHA-inhibitory peptide R18, water or uninjected. The data is combined from two repeated experiments. Fisher’s exact test and pairwise comparison of the percentage of GVBD in the control uninjected cells and control water-injected cells revealed no significant difference between those groups, while a significantly greater percentage of GVBD was noted for oocytes injected with R18 compared to control water-injected cells (* in A). **b** Percent GVBD in oocytes injected with antisense morpholinos against each of the seven mammalian *Ywha* mRNA isoforms as well as the standard nonsense moprholino that was designed to have no effect. The data is combined from two repeated experiments. A significantly greater percentage of GVBD was noted for oocytes injected with morpholino targeting *Ywhah* mRNA compared to control non-sense morpholino (* in B)

In a preliminary experiment to examine YWHA protein function, we injected oocytes with the synthetic YWHA-inhibitory peptide R18. For these experiments, we determined the threshold or critical concentration of dbcAMP, just enough to maintain prophase I arrest in at least 75% of oocytes cultured overnight, to be 0.05 mg/mL. Oocyte maturation experiments in this concentration of dbcAMP permitted us to examine treatments that could promote maturation compared to control cells in which meiotic arrest is maintained but is not dominated by the activation of PKA by dbcAMP. Microinjection of 0.5 mg/mL R18 into oocytes, followed by overnight incubation in media containing 0.05 mg/mL dbcAMP, promoted release from meiotic arrest and GVBD in a larger number of cells compared to uninjected control oocytes or control oocytes injected with deionized water. Ten of 15 (67%) of oocytes underwent GVBD following R-18 injection, while only 4 of 16 (25%) did so when injected with deionized water, and only 5 of 18 (28%) control uninjected oocytes underwent GVBD (Fig. 5a). Examination of the data in the R18 study using Fisher’s exact test and pairwise comparison of the percentage of GVBD in the control uninjected cells and control water-injected cells revealed no significant difference between those groups (*P* = > 0.999). Pairwise comparison of the R18-injected cells to the control water-injected cells revealed a significant difference (*P* < 0.05).

The effectiveness of R18 inhibition is certainly concentration-dependent; however, the injection of a more concentrated peptide solution was not possible. In addition, of course, some residual PKA, kept active by the 0.05 mg/mL dbcAMP, may affect CDC25B as well as other proteins involved in cell cycle control. Nevertheless, the experiment suggests that inhibiting some interactions of YWHA with its binding partners, including CDC25B, shifts the balance toward release from the meiotic arrest. To further investigate this possibility, we chose the following more specific approach of reducing YWHA protein synthesis rather than inhibiting its action.

### Knockdown of YWHA isoforms with antisense morpholinos

In a series of experiments for each YWHA isoform, GV-intact oocytes arrested in prophase I were microinjected with a translation-blocking morpholino oligonucleotide (MO) against mRNA for each of the *Ywha* isoforms at a final intracellular concentration of 0.1 mM. The oocytes were injected with MOs and then held for 24 h in prophase arrest with media containing 0.1 mg/mL dbcAMP to permit a reduction of the existing YWHA protein. The oocytes were then transferred into media containing 0.05 mg/mL dbcAMP, a threshold concentration just sufficient to maintain prophase I arrest, incubated for 14–16 h, and then scored for GVBD (see Fig. 5b). A greater number (70%) of oocytes injected with MO against *Ywhah* mRNA were found to undergo GVBD, in contrast with oocytes injected with MOs targeting other *Ywha* mRNA isoforms. Under identical conditions in this series of experiments, GVBD in control, uninjected oocytes was only about 7.5% and only about 5% for oocytes injected with a standard, nonsense morpholino oligonucleotide known to be incapable of binding to any of the *Ywha* mRNAs. Statistical analysis of 2 × 2 contingency tables using Fisher’s exact test indicated no significance difference in percent GVBD between the uninjected cells and the cells injected with standard nonsense morpholino (*P* > 0.999). Pairwise comparison of each of the *Ywha* isoform morpholino injection conditions with the nonsense morpholino control revealed no significant differences in percent GVBD between the control and the experimental group (*P* > 0.05), with the exception of *Ywhah* (*P* < 0.0001). These results suggested that among the seven mammalian isoforms of YWHA proteins, all of which were found to bind to CDC25B, YWHAH may be important for maintaining the prophase I arrest of mouse oocytes through interaction with CDC25B or other proteins. The experimental analysis is, however, limited since the extent of mRNA inactivation in the knockdown experiments is likely to be variable. Moreover, residual activation of PKA in “threshold” concentrations of dbcAMP may be sufficient to alter oocyte maturation in this experimental situation.

### *Ywhah* or *Ywhae* inactivation by oocyte-specific and global knockouts

To definitively characterize the role of the YWHAH protein isoform, we generated mice with an inactive *Ywhah* gene using the LoxP-Cre system. This permitted generation of animals in which the *Ywhah* gene is inactivated specifically in oocytes or globally in all cells of a female mouse. We also generated oocyte-specific and global *Ywhae* knockout mice, since the YWHAE protein had been suggested by other investigators to play a role in oocyte maturation. The production of these genetically modified mice is described in the Methods section. In brief, oocyte-specific gene inactivation was generated in animals expressing Lox-P sites with ZP3-Cre, a Cre recombinase driven by the zona pellucida protein 3 (ZP3) promoter and which is expressed only in oocytes beginning in primary follicles and maintaining expression in preantral follicles [56]. ACTB-Cre mice were used to generate a global knockout. ACTB-Cre is driven by the human beta actin gene promoter and is expressed in all cells of the embryo by the blastocyst stage [57]. It is expected then, to act on LoxP constructs in early cells of the blastocyst to generate embryos and adults in which gene function is altered in all cells.

### Oocyte-specific *Ywhah* or *Ywhae* gene inactivation does not alter in vivo breeding

**Fig. 6:**
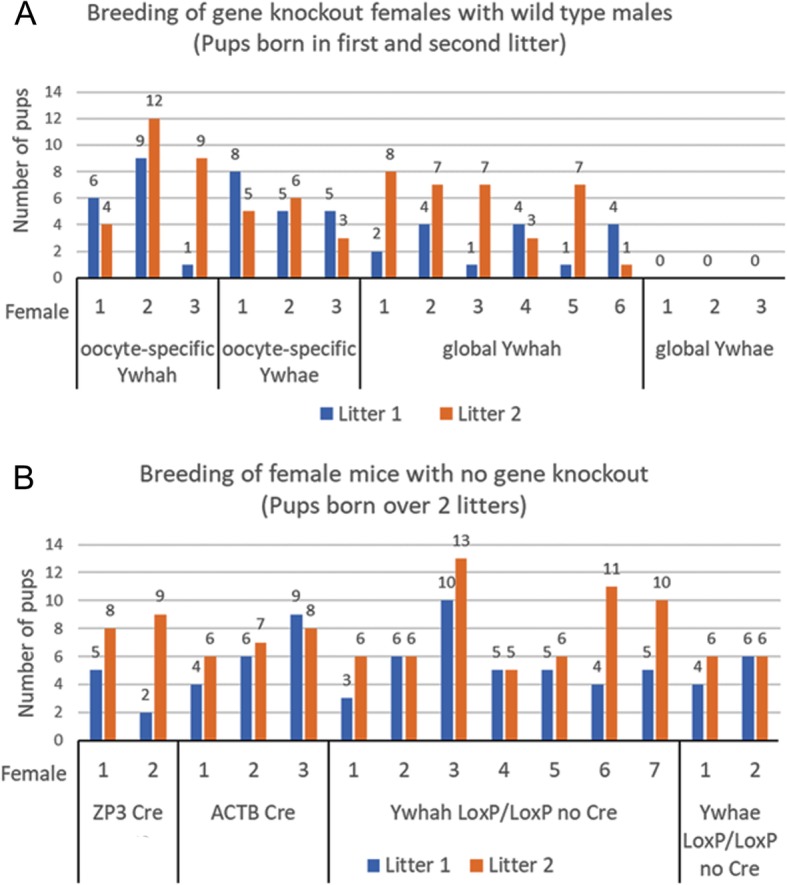
Breeding of *Ywhah* and *Ywhae* oocyte-specific and global gene knockout mice with wild-type males. The number of pups for each female of a given geneotype is indicated for the first two litters. In vivo breding illustrates the range of offspring number in mice of different females mated with different wild-type males. Here, the number of pups produced by females with oocyte-specific inactivation of *Ywhah* or *Ywhae* and global inactivation of *Ywhah* (**a**) falls within the normal range of litter sizes of mice without gene inactivation (here mice containing ZP3 or ACTB Cre, and mice with floxed *Ywhah* or *Ywhae* gene (LoxP/LoxP) and no Cre (**b**). However, females with global inactivation of *Ywhae* did not produce pups (last group in (**a**), see Results and Discussion)

Female mice with oocyte-specific knockout of *Ywhah* or *Ywhae* were mated with wild-type male mice. The females were genotyped to confirm the knockout condition and, when conditions permitted, absence of the gene was also confirmed by Western blot. In vivo breeding indicated that reproductive capacity is not greatly altered with oocyte-specific inactivation of either gene when breeding of pairs were maintained for 2 to 10 months. It should be noted that variation in litter number is common and depends on the characteristics of the female and the male in the breeding pairs and other factors. Females with the oocyte-specific inactivation of *Ywhah* or *Ywhae* are fertile and litter sizes fall in the normal range when compared to litter sizes of females in which *Ywhah* or *Ywhae* genes are not inactivated (mice containing only the floxed alleles for *Ywhah* or *Ywhae* or female mice containing only the transgene for the ZP3 or ACTB Cre recombinase with no modification of the *Ywha* genes). Breeding data of individual females are summarized in Fig. 6.

The litter sizes of females with global inactivation of *Ywhah* in all tissues, including the ovary, are like those of the oocyte-specific knockout animals and are similar to the litter sizes of females in which the gene is not inactivated. In contrast, no offspring are produced when the gene for *Ywhae* is inactivated in all tissues, including the ovary (Fig. 6a, global *Ywhae*). As described below, oocytes from both oocyte-specific and global *Ywhae* knockout mice appear to mature normally. In addition, eggs collected from females with oocyte-specific *Ywhae* inactivation can be fertilized in vitro to produce two-cell embryos without adverse effect on the first meiotic division. Inhibition of in vivo breeding in the global *Ywhae,* but not the oocyte-specific, knockout mouse suggests that YWHAE may be required in somatic cells in other tissues affecting reproductive potential.

### Oocyte-specific or global *Ywhah* or *Ywhae* gene inactivation does not alter oogenesis or in vitro oocyte maturation

**Fig. 7:**
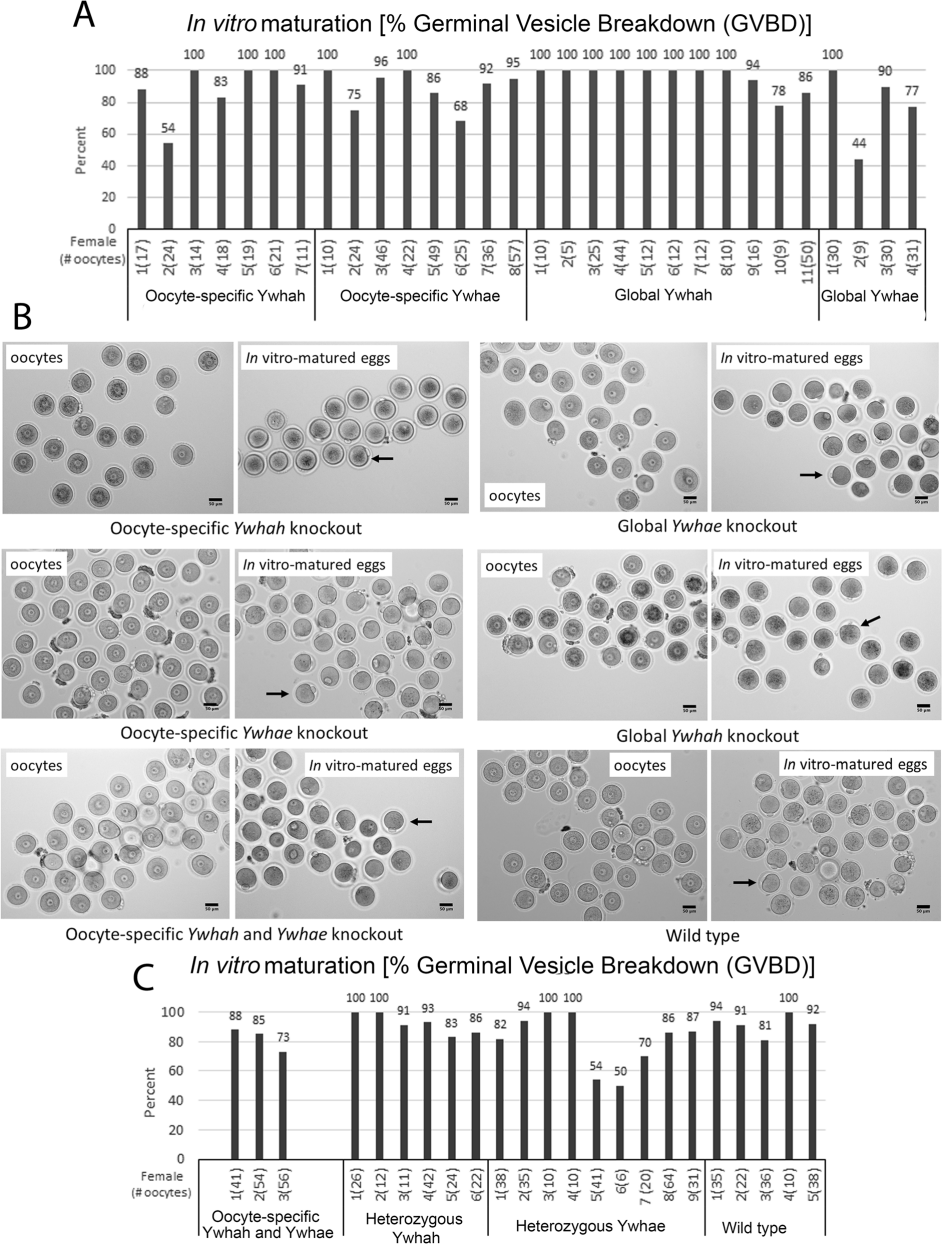
**a** In vitro maturation of oocytes from *Ywhah* and *Ywhae* oocyte-specific and global knockout females. For each female of a given genotype, the total number of oocytes examined is indicated in parentheses and the percent of those oocytes that underwent germinal vesicle breakdown (GVBD) is indicated in the bar graph. The extent of oocyte maturation, as indicated by GVBD, is high in females of all genotypes in which *Ywhah* or *Ywhae* is inactivated specifically in the oocyte or globally. **b** Representative images of oocytes collected from knockout females and the corresponding images of the mature eggs the following day after in vitro maturation of oocytes from knockout females and wild-type females. Oocytes from females of all genotypes appear normal with intact germinal vesicles. Arrows indicate the first polar body of some eggs, which is not always visible because of the orientaion of the egg. Scale bars represent 50 μm. **c** In vitro maturation of oocytes from females in which both the *Ywhah* and *Ywhae* genes were inactivated in the oocyte, oocytes from females in which one allele of *Ywhah* or *Ywhae* was inactivated (hetetrozygous), or oocytes from wild-type females

The in vivo breeding data suggests that normal offspring are produced despite oocyte-specific inactivation of the genes for *Ywhah* or *Ywhae.* To further examine the characteristics of oocytes from these knockout animals we collected oocytes as usual and matured them in vitro to determine if there was any difference in the number of germinal vesicle-intact oocytes collected or any difference the extent of maturation in vitro. Absence of YWHAH or YWHAE protein in oocytes does not appear to affect the number of germinal vesicle-intact oocytes isolated from females primed with eCG and cultured in 3-Isobutyl-1-methylxanthine (IBMX) to prevent spontaneous maturation. The oocytes were of typical size and normal morphology. The extent of in vitro maturation was not altered when oocytes were allowed to mature in media without IBMX. As shown in Fig. 7, the percent of oocytes from a given female that mature is somewhat variable, typically ranging from 75 to 100%. For each female, oocytes cultured overnight in IBMX-free media were classified as having intact GVs or having undergone GVBD indicating oocyte maturation, therefore a non-parametric statistical analysis was done using Fisher’s exact test (2 × 2 contingency table) in which the data for all females for a given group was combined and compared to the data of in vitro maturation of oocytes from wild-type females combined. A two-tailed *P* value of less than 0.05 is considered to indicate a significant difference. Comparing the four knockout conditions to the wild-type data, all pairwise *P* values are greater than 0.05 (oocyte-specific *Ywhah*, *P* = 0.34; oocyte-specific *Ywhae*, *P* = 0.87; global *Ywhah*, *P* = 0.08; global *Ywhae*, *P* = 0.23). When data from the double knockout condition (oocyte-specific *Ywhah* and *Ywhae*) is compared to the wild-type data, the pairwise P value equals 0.05. However, for these three females, maturation of oocytes is mostly normal with the percent of maturation ranging from 73 to 88% (Fig. 7c). Comparing the two heterozygous conditions to the wild-type data, the pairwise P values were greater than 0.5 (heterozygous Ywhahe, *P* = 0.68; heterozygous Ywhae, *P* = 0.015). This suggests that the absence of one allele has no effect on oocyte maturation.

### In vitro fertilization of eggs from mice with oocyte-specific inactivation of *Ywhae is normal*

**Fig. 8:**
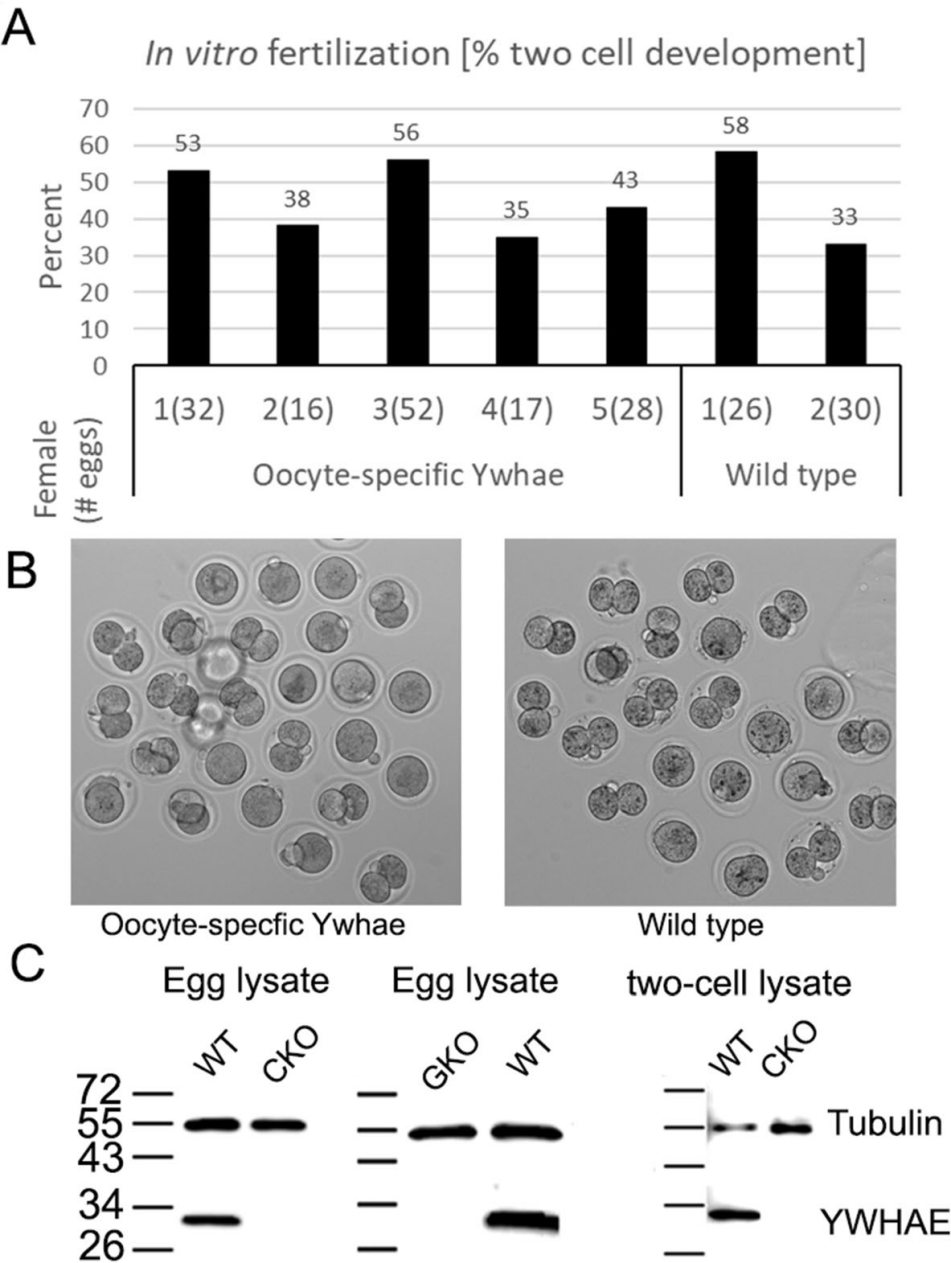
In vitro fertilization of eggs from *Ywhae* knockout females. **a** For each of five different females with oocyte-specific knockout of *Ywhae*, the total number of eggs collected and inseminated is indicated in parentheses and the percent of those eggs that proceeded to the two-cell stage is indicated in the bar graph. Two representative examples of fertilization and percent of two cell development of wild-type eggs are shown. In two cases (females 4 and 5), fertilized eggs from *Ywhae* gene knockout females were continued in culture and developed to the morula and/or blastocyst stage. **b** Representative images of eggs or two-cell embryos following in vitro fertilization of eggs from oocyte-specific *Ywhae* knockout females or a wild-type female. **c** Representative Western blot of cell lysates of eggs collected from wild-type females, lysates of oocyte-specific knockout of *Ywhae* (conditional knockout or CKO), lysates of eggs collected from the global knockout of *Ywhae* (GKO), and lysates of two-cell embryos from fertilized wild-type eggs and from fertilized eggs of females with oocyte-specific knockout of *Ywhae* (conditional knockout or CKO). The blots were probed with antibodies for YWHAE or tubulin (loading control). YWHAE protein was not detected in the knockout eggs nor in the two-cell embryos

To further examine the role of *Ywhae* in mouse oocytes we examined the fertilization potential and early development of eggs collected by superovulation from five different oocyte-specific *Ywhae* knockout females (Fig. 8). Thirty-five to 56% of the eggs from a given *Ywhae* knockout female were fertilized and developed normally to the two-cell stage, which is comparable to typical in vitro fertilization rates of wild-type mice (data from two are shown in Fig. 8). Fertilized eggs from two *Ywhae* knockout females maintained in culture and developed to the morula and/or blastocyst stage.

## Discussion

### Multiple YWHA isoforms are transcribed in mouse oocytes and eggs

All seven *Ywha* isoform transcripts were detected in oocytes from ICR(CD-1) mice by PCR using isoform-specific primers. *Sfn* transcript, while identified in oocytes from ICR(CD-1) mice, was not detected in the inbred C57BL/6 J strain. Single cell mRNA sequencing also revealed transcripts for all seven isoforms in ICR(CD-1) mice and in various knockout models. In contrast, one report indicates that only *Ywhab* and *Ywhae* transcripts could be detected in mouse oocytes [43]. However, several other published reports on the presence of *Ywha* messages in the mouse egg agree with our results. Notably, in an expression profiling study of mature mouse eggs [44], six *Ywha* isoforms were detected (*Sfn* was not listed in this report). In a different analysis of maternal mRNA expression in mature eggs [58] *Ywhab, Ywhag*, *Ywhah*, *Ywhaq* and *Ywhaz* were noted (others were not mentioned in this report which examined only large changes in expression profiles of late one cell embryos compared to eggs). Of additional interest are two reports on human eggs clearly indicating the all seven isoforms of *Ywha* messages exist in the human egg [45, 46]. Taken together, our data on *Ywha* transcripts and protein expression, along with other published results indicates presence of transcripts and proteins of all seven YWHA isoforms in mouse and human oocytes and eggs. Therefore, it is prudent to consider all YWHA isoforms in any analysis of YWHA functional roles in oocyte maturation, fertilization and early embryonic development.

### Exogenously expressed YWHAH and YWHAE co-localize in mouse oocytes and are predominantly cytoplasmic

The exogenous expression of fluorescently labeled YWHAH and YWHAE proteins revealed a distribution of these YWHA proteins like that previously observed by immunofluorescence and immunohistochemistry [42]. The two isoforms are found in similar locations as indicated by a relatively high Pearson’s correlation and both isoforms are found primarily in the cytoplasm and not in the nucleus. It was also previously reported that YWHAE is primarily cytoplasmic in mouse oocytes [43].

### Interactions of YWHA isoforms with CDC25B in oocytes and eggs

Using in situ Proximity Ligation Assays, we have been able to demonstrate that all seven mammalian isoforms of YWHA interact with CDC25B in both mouse oocytes and eggs. This data indicates that the associations of CDC25B with YWHA proteins may occur through interactions with multiple isoforms. It is necessary to examine all YWHA isoforms when examining the regulation of CDC25B. Some previous reports on oocyte and egg YWHA protein interaction with CDC25B have examined only several of the YWHA isoforms [43, 59]. We also note here, that by FRET analysis, that there is a strong interaction between exogenously expressed mouse YWHAH and CDC25B, that suggests binding between the two proteins. We might expect a similar result of FRET analysis in examination of the interaction of CDC25B with other YWHA isoforms.

As we report here for oocytes and eggs, it is known from studies in somatic cells that all seven isoforms bind to CDC25B; however, YWHAB, YWHAG, YWHAH and YWHAQ interact more strongly than YWHAE, YWHAZ and SFN [60]. Moreover, those that bind more strongly cause CDC25B to be exported form the nucleus. While all isoforms of YWHA are capable of binding, the binding of specific YWHA isoforms may affect the localization and function of CDC25B. In addition, it will be important to explore the nature of YWHA heterodimers in regulating maturation. We do not know if YWHA heterodimers form to bind to CDC25B or other target proteins in the oocyte.

### YWHA proteins appear to be important in meiotic arrest of mouse oocytes

Inhibition of YWHA proteins by the inhibitory peptide R18 appears to promote maturation, even in the presence of a threshold concentration of dbcAMP. It has been proposed that YWHA proteins bind to CDC25B and sequester it in the cytoplasm, preventing the premature activation of oocyte maturation. The R18 peptide shares a common binding site on YWHA with other YWHA-binding partners, and peptide R18 competitively inhibits interactions between YWHA and its binding partners [61]; thus, R18 can displace proteins bound to YWHA. The interaction of R18 with YWHA does not depend on the YWHA isoform and the peptide does not require prior phosphorylation for binding. The utility of the R18 peptide has been demonstrated, for example, to show that YWHA is a critical regulator of anti-apoptotic signaling in various cells [62, 63], including cultured ovarian granulosa cells [64]. Introduction of R18 message in frog eggs produced defects in axial patterning and mesoderm specification [65]. Here, when mouse oocytes were microinjected with the synthetic YWHA-inhibitory peptide R18 and incubated in presence of the threshold concentration of 0.05 mg/mL dbcAMP there was a ~ 40% greater incidence of germinal vesicle breakdown compared to uninjected control oocytes or to control oocytes injected with sterile, deionized water. Thus, disruption of YWHA binding to target proteins promotes oocyte maturation.

We utilized isoform-specific morpholino-mediated knockdown experiments which indicated that meiotic arrest in mouse oocytes may be maintained by YWHAH since introduction of this morpholino, targeting *Ywhah* message, appears to be effective in promoting germinal vesicle breakdown in the presence of a threshold concentration of dbcAMP. It has been proposed by other investigators that YWHAE protein is responsible for maintaining prophase I arrest in mouse oocytes, since in one series of experiments, 36% of oocytes underwent GVBD after injection of siRNA to knockdown YWHAE in the presence of dbcAMP [43]. However, injections of siRNA for the other six isoforms were not done by these researchers. We did not find strong evidence for a role of YWHAE in holding eggs in prophase arrest based on the morpholino experiments. Reliance on injection of R18 and inhibitory morpholinos or siRNA is insufficient to define the role of specific YWHA proteins. Introduction of R18 does not mean that all YWHA proteins are inhibited completely. Interference of mRNA by siRNAs or morpholinos does not mean that all protein synthesis is inhibited. By their nature, these are protein knockdown experiments. Moreover, analysis by these methods is complicated by the fact the dbcAMP is present. Multiple proteins are targeted for phosphorylation by PKA. Therefore, we utilized genetic knockout experiments to define the role of the two isoforms that might be important based on our work and others, YWHAH and YWHAE.

### Oocyte-specific *Ywhah* or *Ywhae* gene inactivation does not alter in vivo breeding or in vitro oocyte maturation

If meiotic arrest was solely and completely dependent on YWHAH or YWHAE binding to CDC25B, one might expect that elimination of either of these proteins could lead to premature oocyte maturation, since CDC25B would not be held in the cytoplasm in an inactive state in oocytes within the follicle. We found no evidence that oogenesis was altered when either YWHAH or YWHAE proteins or both were absent. Oocytes obtained from these animals after eCG priming had intact GVs, were normal in size and morphology, and there was no indication of premature meiosis I resumption. Oocytes from these animals underwent normal in vitro maturation. We previously noted that YWHAH may play an important role in meiotic spindle formation by employing antisense morpholino knockdown approaches [55]. The gene knockout experiments presented here suggest that, if YWHA proteins are required in spindle formation, other isoforms might substitute. It would be valuable to follow up on this line of research. Breeding and development of pups was normal in the absence of YWHAH or YWHAE in females with oocyte-specific knockout of these genes.

### Global inactivation of *Ywhah* gene does not alter in vivo breeding or in vitro oocyte maturation

As with oocyte-specific *Ywhah* gene inactivation, we found no apparent difference in fertility, oogenesis or in vitro maturation of cells obtained from animals with global inactivation of the *Ywhah* gene compared to animals containing the gene. This suggests that YWHAH protein is not required in germ cells or in somatic cells for normal reproduction. We found no obvious abnormal phenotype in YWHAH knockout females. This indicates that YWHAH protein is not essential for female reproduction and early embryonic development and, given the importance of YWHA in many cellular events, other isoforms of YWHA might substitute. Normal morphology and presumably normal fertility in female YWHAH knockout mice have also been recently reported by other investigators who found that absence of YWHAH protein leads to deafness and hair cell degeneration [66].

### Global deletion of the *Ywhae* gene prevents breeding without affecting oogenesis, oocyte maturation or in vitro fertilization

No offspring were produced by females when the *Ywhae* gene was inactivated in all tissues, including the ovary and oocytes. However, oocytes collected from these mice appear to mature normally in vitro. With germ-line deletion of *Ywhae* in a global knockout, mice die at birth due to cardiac malformations [48]. Deletion of *Ywhae* in oocytes is apparently compensated by introduction of the normal gene from sperm at fertilization, since apparently normal pups are produced in the oocyte-specific knockout of *Ywhae*, while in the female YWHAE protein may be required in somatic cells in other tissues of the female, for example in the uterus where implantation might be altered. This observation suggests an important role for YWHAE that will need to be examined. It has been reported that only *Ywhae* message is expressed in mouse eggs and that knockdown of *Ywhae* message by small interference RNA inhibits first mitosis in some percentage of fertilized mouse eggs [59]. We find that oocytes collected from females in which *Ywhae* is disrupted in oocytes undergo normal in vitro maturation, in vitro fertilization and early development to the 2-cell stage. Maternal expression of YWHAE protein does not appear to be required for the first mitotic division nor does absence of the protein interfere with the first mitosis.

### YWHA proteins in meiosis

Given the central role of YWHA proteins in regulating the mitotic cell cycle and evidence for regulation of meiosis, additional work is needed to define the roles of the specific isoforms. All YWHA isoforms need examination. Other cell cycle control proteins, in addition to CDC5B, will need to be investigated in more detail to complete the analysis of YWHA proteins in meiotic regulation. Certainly, a number of cell cycle control proteins interact with YWHA, reviewed in [67]. For example, it is known that YWHA proteins interact with Wee1 in frog egg extracts [68], in human somatic cells [27, 69] and with expressed mouse Wee1 protein [70, 71]. Further study will be needed to examine the interaction of YWHAH protein with the oocyte-specific WEE2, its activity and the phosphorylation of CDK1.

## Conclusions

We find that multiple isoforms of YWHA protein are expressed in mouse oocytes and all seven isoforms appear to interact with CDC25B. While this work and other research suggests that YWHA proteins may bind to and regulate the activity of CDC25B, as well as possibly additional proteins that regulate oocyte maturation, the oocyte-specific gene knockout experiments indicate that YWHAH and YWHAE proteins are not required for normal oogenesis, oocyte maturation and early embryonic development. The data suggest that one or several of the other YWHA isoforms may compensate for absence of YWHAH or YWHAE proteins within the oocyte, and this possibility should be explored. Global inactivation of *Ywhah* in female mice does not appear to alter oogenesis, oocyte maturation and early development, while global inactivation of *Ywhae* in female mice prevents breeding without affecting oogenesis, oocyte maturation or in vitro fertilization.

## Methods

### Collection of oocytes and eggs

All mice used in the present experiments were housed and used at Kent State University with the approval of the Kent State University Institutional Animal Care and Use Committee following the appropriate laws, guidelines and policies and performed in accordance with the NIH and National Research Council’s publication, “Guide for Care and Use of Laboratory Animals”. Tissues were only obtained from euthanized mice following the approved protocol. Animals were euthanized by CO_2_ inhalation (approximately 5 min in small container gassed with CO_2_ from a regulated tank of compressed 100% CO_2_). This was followed by cervical dislocation to ensure death. Standard breeding and selection of specific genotypes also included euthanasia of some animals using this protocol. To obtain oocytes, female mice were injected with 5 IU eCG to stimulate follicle growth and 44–48 h later, following euthanasia, the ovaries were removed and repeatedly punctured with a 26-gauge needle to rupture follicles. Cumulus cell-enclosed oocytes were isolated, and the cumulus cells were removed by repeated pipetting though a small-bore pipette. Fully-grown oocytes with intact nuclei (germinal vesicles) were cultured in MEMα supplemented with pyruvate and an antibiotic/antimycotic, and containing 0.1 mg/mL dibutyryl cAMP (dbcAMP; Sigma-Aldrich) or 0.1 mg/mL 3-Isobutyl-1-methylxanthine (IBMX; Sigma-Aldrich), each of which prevents cAMP breakdown and spontaneous oocyte maturation. Mature, metaphase II-arrested eggs were obtained from euthanized mice 13–14 h following superovulation by injection of 5 IU hCG, which was preceded by a priming injection of 5 IU eCG 48 h earlier. The cumulus mass and egg were left intact for in vitro fertilization or the cumulus cells were removed with 0.3 mg/ml hyaluronidase. In some cases, z*onae pellucidae* of oocytes and eggs thus collected were removed by a brief treatment in acidic Tyrode’s solution (Sigma-Aldrich). Oocytes and eggs from outbred mice [ICR(CD-1), Harlan Laboratories, now Envigo] and inbred mice [C57BL/6 J, JAX mice, Jackson Laboratory] were used in these studies as well as transgenic animals, outlined below.

### Messenger RNA isolation and cDNA synthesis for analysis of mRNAs for *Ywha* isoforms

Oocytes and eggs from ICR(CD-1) mice and C57BL/6 J mice were used for mRNA analysis. Messenger RNA was isolated from 50 μL pools of 25 oocyte- and 25 egg- lysates using the Dynabeads mRNA DIRECT™ kit (Thermo Fisher) following the manufacturer’s directions. The mRNA was then eluted off the Oligo (dT)_25_ beads with 10 mM Tris-HCl. First strand complementary DNA stocks were prepared using the QuantiTect Reverse Transcription kit (Qiagen) following the manufacturer’s instructions. Two reaction mixtures of cDNA were made using 5 μL mRNA for each mRNA stock.

Primers were designed for each *Ywha* isoform transcript and for histone H2AfZ (positive PCR control) using the NCBI Primer-BLAST program and were designed according to published mouse mRNA data (see Table 2). All primers were designed to span exon-exon junctions. Amplification of mRNA of the housekeeping gene, H2AfZ, was used as a control. The PCR protocol included 35 cycles of amplification. The PCR products were run on a 2% agarose gel stained with GelRed Nucleic Acid Stain (Phenix) and visualized during exposure to UV light. To confirm the identity of each YWHA isoform, in a parallel experiment, PCR products were sequenced. PCR products were isolated from the residual PCR buffers and primers by DNA Clean and Concentrator − 5 (D4003, Zymo Research) spin columns. The purified PCR DNA template was sequenced by Eurofins MWG Operon using specific primers.

**Table 2:** Primers used to detect the presence of each *Ywha* isoform by RT-PCR

Gene	Forward Primer	Reverse Primer	Product Size
*Ywhab*	5′-AACGATGTGCTGGAGCTGT-3’	5′-CGGATGCAACTTCAGAAAGA-3’	121 bp
*Ywhae*	5′-CAGAACTGGACACGCTGAGT-3’	5′-TTCTGCTCTTCACCATCACC-3’	118 bp
*Ywhah*	5′-CATGAAGGCGGTGACAGA-3’	5′-TAACCCTCCAAGAAGATCGC-3’	110 bp
*Ywhag*	5′-TCCTTCTTTCCAGCCGATCC-3’	5′-GTTCAGCTCGGTCACGTTCTT-3’	139 bp
*Ywhaq*	5′-CGGTGGCCTACAAAAACGTG-3’	5′-ACAATTCCAGGACCGTGGTG-3’	168 bp
*Ywhaz*	5′-CCAGCGACCACCCATTGT-3’	5′-ACGATGACGTCAAACGCTTC-3’	139 bp
*Sfn*	5′-TGTGGCGAAGACTAGGAGGA-3’	5′-GTCTCGAGAGTAACGCTGGG-3’	134 bp
*H2AfZ*	5′-TGCAGCTTGCTATACGTGGA-3’	5′-TCCTTTCTTCCCGATCAGCG-3’	110 bp

### Single cell sequencing for mRNA of *Ywha* isoforms

Single oocytes were isolated in PBS and processed by SingulOmics (New York) for RNA sequencing and data analysis. The computational pipeline for expression quantification was primarily based on STAR aligner and Cufflinks software tool. Reads from RNA-seq were subjected to quality control using FastQC (version 0.11.4), quality trimming using Trim_Galore (version 0.4.1) and aligned to mouse reference genome (GRCM38.91) using STAR (version 020201; options: -outSAMattrIHstart0--outSAMstrandField intronMotif--outFilterIntronMotifs RemoveNoncanonical--alignIntronMin 20--alignIntronMax 1,000,000-outFilterMultimapNmax 1). Duplicated reads were discovered using Picard tools (version 1.119) and removed. Gene annotations (gtf file; version GRCM38.91) were obtained from Ensembl. FPKM values of genes were estimated using cufflinks (version 2.2.1). FPKM values give some qualitative and quantitative estimate of gene expression. Data was obtained for oocytes collected from females of the wild type, ICR(CD1). Oocytes were also obtained from the oocyte-specific knockouts of *Ywhae* and *Ywhah*, and the oocyte-specific double knockout for *Ywhae* and *Ywhah*. Additionally, we examined an oocyte from a female in which the *Ppp1CC* gene is globally inactivated in mice with a ICR(CD-1) background; this oocyte provided one additional cell for comparison. Absence of the *Pppp1CC* does not appear to reduce female fertility. This line has been described previously and is well studied [72, 73].

### Microinjection of RNA constructs mCherry-YWHAH, EGFP-YWHAE and mCherry-CDC25B

Messenger RNA constructs were designed and analyzed using SnapGene Viewer. Sequences for EGFP (717 bp) or mCherry (708 pb) fluorescent probes were added to the N-terminal sequences of mouse *Ywhah* (741 bp), *Ywhae* (768 bp) or Cdc25b (variant 1, 1731 pb). A 3xGGGGS (Glycine and Serine residue) linker was added between the fluorescent probe and the target sequence to provide flexibility for fusing of the two proteins. The mRNA was synthesized by TriLink Biotechnologies (San Diego, CA) and the final product included the company’s proprietary 3′ CleanCap®. The concentration and quality of final the product was determined by quality control and gel analysis. Oocytes were injected using a semi-quantitative pneumatic pressure injection system described previously [55]. Approximately 10 pL of 25 μg/mL mRNA was injected into the cytoplasm of each GV-intact mouse oocyte, giving a final concentration of approximately 1.25 ng/μL mRNA, assuming an oocyte volume of 200 pL. The injection solution contained mRNA for each of the proteins examined individually or in specific combination at the same final concentration of each for co-localization and FRET analyses. The injected oocytes were incubated in HEPES-buffered MEM with dbcAMP in a humidified chamber at 37 °C and examined the following day. All injected cells display strong fluorescence of EGFP and mCherry-labeled proteins.

### Immunofluorescence

To examine the initial stages of oocyte maturation and the distribution of CDC25B, oocytes were isolated in MEM containing 0.1 mg/mL dbcAMP and examined by confocal immunofluorescence microscopy using a rabbit anti-CDC25B. Cells were fixed immediately in 3.7% formaldehyde (Time 0; 7 cells), or fixed at 30 min (12 cells), 1 h (8 cells) and 2 h (6 cells) after transfer to MEMα containing no dbcAMP. After fixation, the oocytes and eggs were washed in PBS-PVA (PBS containing 1% PVA), permeabilized with 1% Triton X-100 to promote antibody penetration, washed in PBS-PVA, treated with blocking buffer (5% normal donkey serum) and incubated overnight with primary antibody. Following washing in blocking buffer (1% donkey serum), the cells were incubated with appropriate secondary antibody for several hours, washed again, and individually imaged. The primary antibody used in the time course assay was rabbit anti-CDC25B (Proteintech; #10644–1-AP) diluted 1:200, and the secondary antibody used was donkey anti-rabbit (549 nm-conjugated; Jackson ImmunoResearch Laboratories) diluted 1:200. Dilutions were made in 1% donkey serum blocking buffer. Three control oocytes were processed through identical procedure stated above but without incubation in the primary antibody.

### In situ proximity ligation assays (PLA) to detect interaction of YWHA isoforms with CDC25B in mouse oocytes and eggs

Duolink in situ PLA probes, blocking solution, antibody diluents, wash buffers A and B, and detection reagents were obtained from OLink Bioscience (currently distributed by Sigma-Aldrich). Following fixation and permeabilization, the cells were processed following the manufacturer’s instructions for the Duolink in situ PLA kit as previously described [55] with primary antibodies for the YWHA isoforms (rabbit anti-14-3-3 isoform panel PAN017, AbD Serotec; diluted 1:200 in Duolink in situ antibody diluent) and with the primary antibody for CDC25B (goat anti-CDC25B, Santa Cruz, sc-6948; diluted 1:200 in Duolink in situ antibody diluent). Confocal Z-stack images of cells were captured at 3 μm thickness intervals, with a 60X oil immersion lens at a zoom setting of 2.5 for multiple oocytes and eggs for each isoform tested. The experiments utilized a panel of antibodies (#PAN017; AbD Serotec) that was shown to be specific for the various YWHA isoforms in a variety of cell types [42, 74–76]. The goat anti-CDC25B antibody used in these experiments appears to be specific as it co-localized with a rabbit anti-CDC25B antibody directed at a different epitope on the same protein, and with a phospho-specific anti-mouse CDC25B antibody. Control oocytes and eggs were processed simultaneously for in situ PLA following identical procedure in absence of the primary antibodies.

To confirm that the PLA method was effective in identifying the close proximity of two different antibodies under the conditions used in these experiments, three oocytes were processed for in situ PLA using two different antibodies to CDC25B. In this case, each of the antibodies was targeted to the same protein. The first was an antibody raised in goat against a peptide mapping at the N-terminus of CDC25B of human origin, which reacts also with mouse CDC25B. This antibody was used in studying YWHA and CDC25B intractions, and the second antibody was made in rabbit against a different epitope of CDC25B of human origin, which is known to react with mouse CDC25B. Three control oocytes were processed simultaneously for in situ PLA following identical procedure in absence of the primary antibodies.

### Förster resonance energy transfer (FRET) by sensitized emission

ICR(CD-1) oocytes were co-injected with mRNA for two fluorescently labeled proteins and cultured overnight, as indicated above. Cells microinjected with mRNA encoding donor (EGFP) protein or acceptor (mCherry) protein were imaged separately to account for donor and acceptor spectral bleed-through. However, for this FRET pair there is little bleed-through. Olympus FV software was used for the analysis of FRET interactions between mCherry-YWHAH and EGFP-CDC25B, and between EGFP-YWHAE and mCherry-YWHAH. Twelve oocytes were examined for YWHAH and CDC25B interactions and five oocytes for the interaction between YWHAH and YWHAE.

### Microinjection of R18

R18 peptide (Enzo Life Sciences), dissolved in sterile deionized water, was microinjected as described above, into ICR(CD-1) mouse oocytes, to inhibit interactions of all isoforms of YWHA with their potential binding partners including CDC25B. Ten pL of a 10 mg/mL stock solution of R18 (approximately 5% of the oocyte cell volume) was injected into the cytoplasm of the oocyte. Typically, a concentration of 0.1 mg/mL dibutyryl cAMP (dbcAMP) in the oocyte collection medium is enough to maintain the meiotic arrest in isolated mouse oocytes. We determined the threshold or critical concentration of dbcAMP, just sufficient to maintain prophase I arrest in at least 75% of oocytes cultured overnight, to be 0.05 mg/mL. Following injection, the cells were transferred through several washes and incubated in bicarbonate buffered MEMα containing the threshold concentration of dbcAMP in the presence of 5% CO_2_ at 37 °C for 13 h and then examined. Controls included oocytes processed simultaneously through identical procedure, following injection of 10 pL sterile deionized water or with no injection.

### Microinjection of translation-blocking morpholinos against messages for *Ywha* isoforms

GV-intact ICR(CD-1) oocytes were isolated and microinjected, as described above, with 10 pL of 2 mM stock morpholino oligonucleotide (Gene Tools; final concentration 0.1 mM in each cell) against each of the seven *Ywha* isoform messages. The cells were then incubated for 24 h in bicarbonate-buffered MEMα containing dbcAMP (0.1 mg/mL) to prevent maturation and to allow degradation of the existing proteins. The cells were then transferred through several washes and incubated in bicarbonate-buffered MEMα containing 0.05 mg/mL dbcAMP in the presence of 5% CO_2_ at 37 °C for 13 h and examined for intact GV or evidence of GVBD. The number of cells with completed GVBD after injection of morpholino oligonucleotide against each of the *Ywha* isoform messages was compared to the number of cells with GVBD in control uninjected cells and those injected with a standard, nonsense morpholino.

For experiments involving microinjections of R18 and morpholinos against *Ywha* isoform messages, at each injection condition the cells were classified as having intact GV or having undergone GVBD; therefore, a non-parametric statistical analysis was done using Fisher’s exact test (2 × 2 contingency table). First, uninjected cells and control injected cells (deionized water for R18 experiments and a standard, nonsense morpholino for the morpholino experiments) were compared. Then, each experimental condition was compared to the control injection condition (water or the nonsense morpholino). Two-tailed *P* values are presented and a value of less than 0.05 is considered to indicate a significant difference.

### Conditional and global knockouts for *Ywhah* and *Ywhae* isoforms

**Fig. 9:**
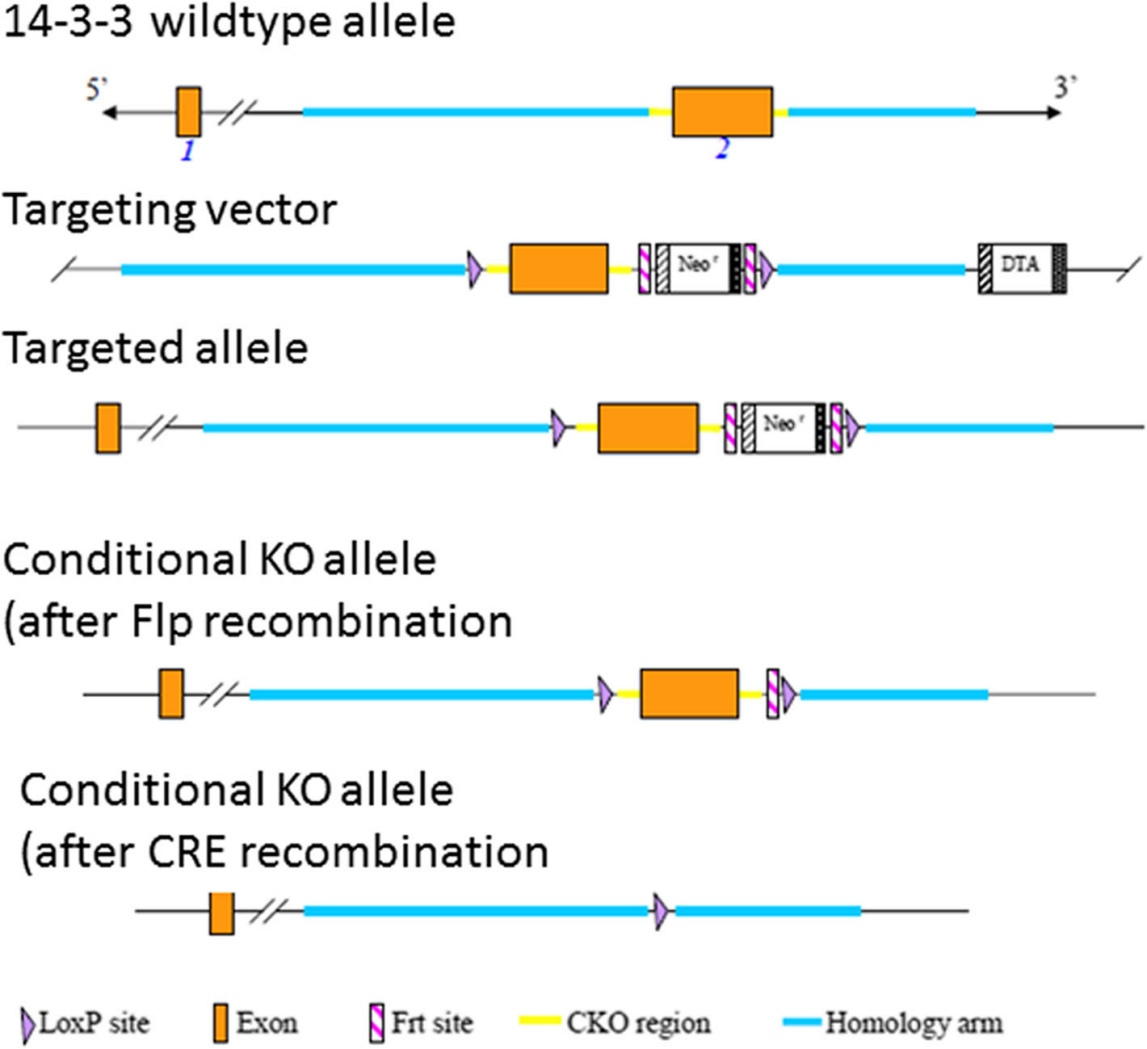
Diagrammatic summary of the production of *Ywhah* oocyte-specific conditional knockout. Exons 1 and 2 of the *Ywhah* gene are represented by orange boxes. The targeting vector and homology arms are indicated. The vector was electroporated into C57BL/6 ES cells, chimeras were produced, and germ line transmission in C57BL/6 J was confirmed. Exon 2 is removed following Cre recombination (see Methods)

We produced a mouse line in which exon 2 of the mouse *Ywhah* gene was flanked by LoxP sites. The conditional knockout mouse line was produced by a commercial company (Cyagen Biosciences, Santa Clara, CA) using standard ES cell-based gene targeting. Exon 2 of the mouse *Ywhah* gene was selected as the conditional knockout region (see Fig. 9). The targeting vector, 5′ homology arm, 3′ homology arm and CKO regions were amplified from BAC DNA and confirmed by end sequencing. The targeting vector was electroporated into C57BL/6 ES cells. Clones were screened by PCR and selected by Southern blot. ES cells were injected into B6-albino mice blastocyts and the chimera produced were bred for germline transmission. Progeny containing the floxed gene were mated with C57BL/6 J mice to obtain a colony on this strain. Mice homozygous for the LoxP in the *Ywhah* gene were mated with ZP3-Cre (C57BL/6-Tg(Zp3-cre), The Jackson Laboratory) or ACTB-Cre mice (B6N.FVB-*Tmem163*^*Tg(ACTB-cre)2Mrt*^/CjDswJ, The Jackson Laboratory). Progeny of these mating pairs were examined by PCR to show disruption of the gene and/or by protein analysis to show loss of protein. These mice were used for in vivo breeding, oocyte analyses, oocyte maturation and in some cases, for in vitro fertilization studies.

We also obtained a mouse line with LoxP sites inserted in the *Ywhae* gene from Dr. K. Toyo-oka (Drexel University). These mice contain LoxP sites to remove exons 3 and 4 of the *Ywhae* gene with Cre recombinase and have been successfully used to study the role of YWHAE protein in the developing brain [51, 77]. Mice were maintained in the floxed condition in 129/SvEv mice and animals homozygous for the LoxP in the *Ywhae* gene were mated with ZP3-Cre or ACTB-Cre mice. We also cross bred both LoxP *Ywhah* and *Ywhae* lines to get a homozygous mouse line for both genes and mated those mice with the ZP3-Cre line to knockout both genes.

Unique primers were used in PCR to confirm disruption of the *Ywhah* and *Ywhae* genes (see Table 3) using DNA from ear-punches or, in some cases, from oocytes. The *Ywhah* LoxP primer distinguished the mice that carried the flanked gene from the wild type. The size for the flanked gene is 292 bp, while the wild-type gene is 226 bp. Heterozygous LoxP animals expressed two different bands with two different sizes: one for the wild type and another for the flanked gene. Absence of any band indicated disruption of the gene. In addition, primers external to the gene knockout region were used to confirm the knockout condition, producing a band at 390 bp. The *Ywhae* LoxP primer distinguished the mice that carry the flanked gene from the wild type condition. The band-size for the flanked gene is 536 bp, while the wild-type gene is 450 bp. The heterozygous condition would express two different bands at two different sizes: one for the wild-type and the other for the flanked gene. Absence of any band indicated disruption of the gene. In addition, primers external to the gene knockout region were used to confirm the knockout condition, producing a band at 664 bp. A generic Cre primer set detected the sequence of the Cre that was found in both ZP3 and ACTB lines. The band at 100 bp confirms the presence of the Cre.

**Table 3:** Primers used to detect LoxP and *Ywhah*

PCR product	PCR primers	Fragment size (bp)
*Ywhah* LoxP	Forward: 5′- TAATTGTGAGCCACCCGAAATGA − 3′ Reverse: 5′- GCCAACGACCAATGCCAATTATAG − 3′	WT: 226 Floxed: 292
*Ywhah* knockout	Forward: 5′- CCTGATCTAGGATAGCTAGGGCTACATAG − 3′ Reverse: 5′- AGTATACCTTTTGGAGACAGGATCTATTATAGCC − 3′	Deletion gives band at: 390
*Ywhae* LoxP	Forward: 5′- GCATGTGTTTGTCTGTCAGAGGAC − 3′ Reverse: 5′- AGGTACCAAAACAGTAAGCCATCTCCCTA − 3′	WT: 450 Floxed: 536
*Ywhae* knockout	Forward: 5′- TTCTTTTGTAGAAATTGGGGAAGGTCATGG − 3′ Reverse: 5′- AGGTACCAAAACAGTAAGCCATCTCCCTA − 3′	Deletion gives band at: 664
Generic Cre	Forward: 5′- GCG GTC TGG CAG TAA AAA CTA TC − 3′ Reverse: 5′- GTG AAA CAG CAT TGC TGT CAC TT − 3′	Cre Positive: 100

Homozygous WT, homozygous LoxP with no Cre and LoxP/WT with no Cre, are effectively wild-type females, which served as control animals in some cases (generally litter mates) along with true wild-type animals. LoxP/WT with Cre positive, −/WT, −/LoxP with Cre negative and WT/LoxP with ZP3 Cre positive were taken as heterozygous females. The ZP3 conditional knockout expressed −/LoxP with ZP3 Cre positive in ear-punch while the oocytes do not express the gene. The global knockout females were homozygous for the knockout (−/−) in all cells, as identified by the primers listed in Table 3.

### Western blotting for knockout mice

In several cases, we confirmed the knockout condition in ovary or oocyte cell lysates using specific antibodies in Western blots. Cell lysates were prepared from oocytes, eggs and 2-cell embryos in a homogenizing buffer (Tris-HCl 10 mM pH 7.0, EGTA 1 mM and EDTA 1 mM pH 8.0, Protease and Phosphatase Inhibitor Cocktail (Sigma-Aldrich # PPC1010). Laemmli sample buffer (5X, containing Tris-HCl pH 6.8–7.0, glycerol 50%, SDS 5%, bromophenol blue 0.05% and DTT 250 mM) was then added and the lysate was boiled for 5 min at 95 °C. Cell lysates were separated in a 12% polyacrylamide gel and the proteins were transferred by electrophoresis to a PVDF membrane (Amersham Hybond P 0.2 PVDF 10600021) and blocked with 5% non-fat dry milk in TTBS (0.2 M Tris, pH 7.4, 1.5 M NaCl, 0.1% thimerosal and 0.5% Tween 20). The blots were incubated with primary antibody overnight at 4 °C [YWHAH: mouse monoclonal anti-YWHAH (Novus Biological, NBP1–92691, 1:5000 dilution) or goat anti-YWHAH (R&D Systems AF4420, 1:1000 dilution); YWHAE: anti-YWHAE (Santa Cruz, sc-23,957, 1:1000 dilution)]. The blots were then washed with TTBS twice for 15 min each and incubated with the appropriate secondary antibody conjugated to horseradish peroxidase for at least 1 h at room temperature. The blots were then washed with TTBS twice for 15 min each and twice for 5 min each. Finally, the blots were developed with Amersham ECL Prime Western Blotting Detection Reagent (Amersham, RPN2232) and imaged.

#### In vitro fertilization

Male reproductive tissue was removed from male mice following euthanasia (as described above for the collection of oocytes and eggs). Sperm were collected by puncture and snipping of each *cauda epididymis* and *vas deferens* of three- to five-month old wild-type male mice in a 1000 μL drop of HTF media (Millipore: EmbryoMax® Human Tubal Fluid) and cultured at 37 °C under 5% CO_2_ in a humidified incubator for 10 min to allow the sperm to swim out. The tissues were removed from the drop after 10 min and sperm were maintained in the incubator for 45 min to allow for capacitation. Eggs were obtained from two- to three-month old wild type and *Ywhae* oocyte-specific knockout females following superovulation induced by intraperitoneal injection of 5 IU of eCG (Sigma-Aldrich) and 46–50 h later, injection of 5 IU of hCG (Sigma-Aldrich). Oocyte/cumulus complexes were removed from the ampullae 13–15 h after hCG injection and transferred to a 135 μl drop of HTF covered with mineral oil. After capacitation, 15 μL of the sperm suspension was added to the 135 μL drop of containing oocyte/cumulus complexes. The dish was incubated at 37 °C in 5% CO_2_ for 4 h. The eggs were then washed by pipetting into fresh media (removing dissociated cumulus cells) and incubated overnight in a 300 μL drop of HTF medium at 37 °C and 5% CO_2_. The following day, the number of two-cell embryos was counted. Some two-cell embryos were cultured to blastocyst stage.

## Data Availability

All data sets used and/or analyzed during the current study are available from the corresponding author on reasonable request.

## Abbreviations

CDC25B: Cell division cycle 25B
dbcAMP: Dibutyryl cyclic adenosine monophosphate
eCG: Equine Chorionic Gonadotropin
FITC: Fluorescein Isothiocyanate
GV: Germinal Vesicle
GVBD: Germinal Vesicle Breakdown
hCG: Human Chorionic Gonadotropin
HTF: Human Tubal Fluid (media)
IBMX: 3-Isobutyl-1-methylxanthine
MEM: Minimal Essential Medium
PKA: Protein Kinase A
PLA: Proximity Ligation Assay
PMSF: Phenylmethylsulfonyl fluoride
R18: YWHA inhibitor peptide
SFN: Stratifin (14-3-3σ)
Tris-HCl: Tris-Hydrochloride
TTBS: Tris Buffered Saline with Tween 20
YWHA: Tyrosine 3-Monooxygenase/Tryptophan 5-Monooxygenase Activation protein (14-3-3) YWHAB: Tyrosine 3-Monooxygenase/Tryptophan 5-Monooxygenase Activation protein, Beta polypeptide (14-3-3β)
YWHAE: Tyrosine 3-Monooxygenase/Tryptophan 5-Monooxygenase Activation protein, Epsilon polypeptide (14-3-3ε)
YWHAG: Tyrosine 3-Monooxygenase/Tryptophan 5-Monooxygenase Activation protein, Gamma polypeptide (14-3-3γ)
YWHAH: Tyrosine 3-Monooxygenase/Tryptophan 5-Monooxygenase Activation protein, Eta polypeptide (14-3-3η)
YWHAQ: Tyrosine 3-Monooxygenase/Tryptophan 5-Monooxygenase Activation protein, Theta/Tau polypeptide (14-3-3τ)
YWHAZ: Tyrosine 3-Monooxygenase/Tryptophan 5-Monooxygenase Activation protein, Zeta polypeptide (14-3-3ζ)
